# Effect of the Brazilian Conditional Cash Transfer and Primary Health Care Programs on the New Case Detection Rate of Leprosy

**DOI:** 10.1371/journal.pntd.0003357

**Published:** 2014-11-20

**Authors:** Joilda Silva Nery, Susan Martins Pereira, Davide Rasella, Maria Lúcia Fernandes Penna, Rosana Aquino, Laura Cunha Rodrigues, Mauricio Lima Barreto, Gerson Oliveira Penna

**Affiliations:** 1 Federal University of Bahia, Institute of Collective Health, Salvador, Bahia, Brazil; 2 Fluminense Federal University, Health Sciences Center, Institute of Community Health, Niterói, Rio de Janeiro, Brazil; 3 London School of Hygiene and Tropical Medicine, Department Infectious and Tropical Diseases, London, United Kingdom; 4 University of Brasília, Tropical Medicine Center, Brasília, Distrito Federal, Brazil; Kwame Nkrumah University of Science and Technology (KNUST) School of Medical Sciences, Ghana

## Abstract

**Background:**

Social determinants can affect the transmission of leprosy and its progression to disease. Not much is known about the effectiveness of welfare and primary health care policies on the reduction of leprosy occurrence. The aim of this study is to evaluate the impact of the Brazilian cash transfer (Bolsa Família Program-BFP) and primary health care (Family Health Program-FHP) programs on new case detection rate of leprosy.

**Methodology/Principal Findings:**

We conducted the study with a mixed ecological design, a combination of an ecological multiple-group and time-trend design in the period 2004–2011 with the Brazilian municipalities as unit of analysis. The main independent variables were the BFP and FHP coverage at the municipal level and the outcome was new case detection rate of leprosy. Leprosy new cases, BFP and FHP coverage, population and other relevant socio-demographic covariates were obtained from national databases. We used fixed-effects negative binomial models for panel data adjusted for relevant socio-demographic covariates. A total of 1,358 municipalities were included in the analysis. In the studied period, while the municipal coverage of BFP and FHP increased, the new case detection rate of leprosy decreased. Leprosy new case detection rate was significantly reduced in municipalities with consolidated BFP coverage (Risk Ratio 0.79; 95% CI  = 0.74–0.83) and significantly increased in municipalities with FHP coverage in the medium (72–95%) (Risk Ratio 1.05; 95% CI  = 1.02–1.09) and higher coverage tertiles (>95%) (Risk Ratio 1.12; 95% CI  = 1.08–1.17).

**Conclusions:**

At the same time the Family Health Program had been effective in increasing the new case detection rate of leprosy in Brazil, the Bolsa Família Program was associated with a reduction of the new case detection rate of leprosy that we propose reflects a reduction in leprosy incidence.

## Author Summary

Leprosy is considered a poverty related disease. Not much is known about the effectiveness of welfare and primary health care policies on reduction of leprosy occurrence. We conducted a study to evaluate the impact of the Brazilian conditional cash transfer (Bolsa Família Program) and the Primary Health Care (Family Health Program) on the new case detection rate of leprosy in the period 2004–2011 in the Brazilian municipalities. All variables were obtained from national databases and a total of 1,358 municipalities were included in the analysis. The new case detection rate of leprosy was significantly reduced in municipalities with intermediate, high, and consolidated BFP coverage. There was a significant increase in new case detection rate of leprosy as Family Health Program coverage increased. We interpret this to mean that at the same time the primary health care had been effective increasing the new case detection rate of leprosy in Brazil, there is an impact of conditional cash transfer in the reduction of the new case detection rate of leprosy due to reduction in leprosy incidence. We expect that these results contribute with arguments to the discussion on the relationship between distributive social policies and health conditions of the population in developing countries worldwide.

## Introduction

According to WHO “leprosy is a chronic infectious disease caused by *Mycobacterium leprae*”. It can lead to physical disability, social stigma and suffering. Although significant improvements have been achieved in disease control, leprosy remains a public health problem in many countries with high incidence and transmission, mainly in tropical Africa, the Indian subcontinent, Pacific and Indian Ocean Islands and South America [1], [2].

The new case detection rate (NCDR) of leprosy remains high in several parts of the world, including Brazil, although the known prevalence in the world has been reduced [3]. In area and population Brazil is the largest country in South America, and the fifth largest in the world. It has the highest leprosy occurrence in the American continent. The country contributed with 16% of new cases detected worldwide in 2011 [2].

Leprosy cases are concentrated in the poorest regions of the country, especially the North, Middle West and Northeast [3], with the last region having the highest proportion of families receiving and benefiting from social programmes such as Bolsa Família Program (BFP) [4], [5]. In 2012 the overall known prevalence of leprosy in Brazil was 1.5 per 10,000 (equivalent to 29,311 individuals in treatment) and the new case detection rate (NCDR) was 17.2 per 100,000 (33,303 new cases) [6].

The known leprosy prevalence is calculated from the number of patients in treatment in a population reflecting the total patients in the moment of the analysis. It is related to the quality of treatment and the time that patients remain with active record in the health system. Paucibacilary patients remains in treatment for 6 months and multibacilary for 12 unless there are complications [7]. Hidden prevalence includes undiagnosed cases (which are mainly responsible for transmission of the leprosy). The NCDR of leprosy which reflects the incidence is calculated from the number of new cases detected in a given population [7], [8]. Because the average time in treatment in less than one year, the known prevalence should be lower than NCDR.

Leprosy is a disease of poverty. Key risk factors reported to be associated with leprosy are crowding, low educational level, lack of hygiene, social inequality, food shortage and malnutrition [9], [10], [11], [12]. It is not clear which influences the risk of infection and which influences the risk of evolution from infection to disease.

Historically, the decline in leprosy is likely to have resulted from socioeconomic development: leprosy started to decline in Spain [13] and disappeared from Japan [14] and Norway [15] before implementation of the WHO multi-drug strategy. The disappearance in Hawaii, was attributed to economic development influencing family crowding, schooling, and nutritional status and others factors [16], [17]. Chabot *et al.* (1995) [18], argued that economic crisis had a negative impact on health care and on poverty related diseases in Africa, including leprosy. Furthermore, economic, political, demographic and social changes in Brazil during the last 40 years had a clear impact on social determinants of Brazilians' health [19]. During this period, there was an expansion of programs and activities in education, health, employment, housing, social security and social development [20]. This probably contributed to the reduction of infectious diseases but it is not clear how this affected leprosy in country.

Conditional cash transfer programs are strategies that have increasingly garnered attention as a means to reduce poverty and inequalities in low and middle-income countries. These programs provide an income for poor families if they comply with specific conditions in education and health [21]. Cash transfers can significantly increase household consumption, reduce food insecurity, increase school enrollment and retention and improve health and nutritional outcomes under certain conditions [22].

Literature on cash transfer programs and their impact on leprosy is currently non-existent. However, recently evidence of this effect has been shown for HIV prevention programmes and other sexually transmitted diseases in underdeveloped countries [23], [24]. Other studies discuss the positive effect of socio economic interventions, like cash transfer programs, in strengthening tuberculosis control by improving household's living conditions and therefore decreasing the exposure to biological risk factors (such as malnutrition) leading to better access and variety to food and health-seeking behavior thus reducing people's vulnerability to infection and disease [25], [26].

The “Bolsa Família” Program (BFP), introduced in Brazil in 2003, was aimed at families in poverty and extreme poverty. It has three main objectives: to transfer income (promoting an immediate relief of poverty), to improve access to education and health care and to offer complementary social programs (enabling families to end their condition of vulnerability) [27]. BFP is the largest cash transfer program in the world with 13.7 million families benefiting in 2012. At the time the program aimed to transfer cash to those defined as “extremely poor families” with monthly per capita income $35 or less and “poor families” (monthly per capita income between $35 and $70 and with children 17 years old or younger or pregnant or lactating women) after enrollment in register of social programs (CadÙnico, in Portuguese). Benefits range from $18 to $175 per month [28]. Enrolled families have to meet education and health conditions of BFP (education and health conditionalities): up to date vaccination, nutritional surveillance of children under 7 years, attendance to ante natal care by pregnant women and post natal care after delivery [29]. It is well established that BFP reduces extreme poverty and contributed to mitigating the social and economic inequalities in Brazil [30], [31]. The observed effect is explained by increased income, improves the food consumption and supplies related to health among the poor and extremely poor individuals [28].

The Family Health Program (FHP), was introduced in 1994, and contributed to the expansion of the Unified National Health System (SUS). SUS principles include decentralization, universality and equity. According to the programme guide: “The FHP is a nationwide program, aimed at broadening access to public health services, especially in deprived areas, by offering free community based primary care” [32]. By 2013, the program was implemented in 96% of Brazil's municipalities, covering 56.4% of the national population [33].

The FHP is widely decentralized and is managed, following national regulations, at the municipality level. It consists of multiprofessional teams with physicians, nurses, community health agents, oral health agents and dentists. Each FHP team is responsible for a well defined population, within an area, with systematic visits, to deliver health care, promotion and prevention. Actions include prenatal, neonatal and under-5 care, immunization and, more relevant for this analysis, prevention, and management of infectious diseases [32]. FHP contributes to leprosy control by supporting early detection and treatment of cases, contact tracing, control of disabilities and other preventive measures [34]. Increased access to primary care (PHC) achieved in Brazil mainly by FHP implementation has been shown to increase new case detection rate of leprosy [35].

There is clear evidence of the effectiveness of BFP and FHP in reducing malnutrition, childhood mortality, and other outcomes related to maternal and child health [36], [37], [38], [39], [40]. The objective of this study is to evaluate the impact of the Bolsa Família Program and Family Health Program on new case detection rate of leprosy in Brazil during the period 2004–2011.

## Methods

### Design, site and study period

A study with a mixed ecological design, a combination of an ecological multiple-group and time-trend study design was carried out, with the municipality as unit of analysis, over the period from 2004 to 2011. Of the 5,570 Brazilian municipalities 1,358 were selected because they belong to high risk *clusters* for leprosy detection previously described [3], [41].

The annual new case detection rate of leprosy (NCDR), was calculated as the number of reported new cases of leprosy (defined by the code A30 in the International Classification of Diseases - 10th revision), per 100,000 people [33].

There are two possible indicators of BFP coverage from the number of families in the program: a) Coverage of target population (poor and extremely poor) was obtained from Ministry of Social Development database. It is defined as “number of families included in the program by municipality divided by the number of eligible families (according to BFP criteria) in the same municipality” [42] and b) Coverage of total population was defined as: “number of individuals enrolled in the BFP (obtained by multiplying the number of beneficiary families by the average family size) divided by the total population of the same municipality” [40].

The indicators obtained were combined and four categories were created according to the tertiles of the distribution of BFP coverage in the total population: low (BFP coverage of the total population of the municipality from 0.0 to 27.75%), intermediate (27.76–48.10%), high (> = 48.11%) and consolidated (BFP coverage of the total population of the municipality>48.11% in the presence of BFP coverage of the target population ≥100% for at least the last 4 years).

The yearly coverage of the FHP was calculated as the number of individuals with records in any of the FHP facilities of the municipality in that year divided by the population of the municipality [43]. FHP coverage was categorized according to tertiles of the distribution (1st tertile: 0–72.02%, 2 st tertile: 72.03–95.06% and 3 st tertile: over 95.06%).

A group of covariates was selected as potential leprosy determinants based on the literature [9]: percent of the population younger than 15 years, illiteracy rate, unemployment rate, urbanization rate, average number of residents per household, percentage of poor people in the city (proportion of individuals with per capita household income equal to or less than U$ 35,00 monthly) and Gini Index that is a measure of income distribution.

Gini Index is defined as “measures the extent to which the distribution of income or consumption expenditure among individuals or households within an economy deviates from a perfectly equal distribution. A Gini index of 0 represents perfect equality, while an index of 100 implies perfect inequality” [44]. We dichotomized the covariates according to the median value of their distribution.

### Data sources

The data used were collected from different information systems:

Leprosy NCDR: the Notifiable Diseases Information System (SINAN) of the Ministry of Health [33].

BFP coverage: the Ministry of Social Development database [42].

FHP coverage: the Primary Care Information System (SIAB) [33].

Population and Socioeconomic variables: The Brazilian Institute of Geography and Statistics [45].

Some variables were extracted from the 2000 and 2010 national demographic census databases; in these cases, values for 2004–09 were estimated by linear interpolation and by linear extrapolation for 2011.

### Statistical analyses

A descriptive analysis to describe trends in mean BFP and FHP coverage and in the variables. We measured the impact of BFP and FHP on the NCDR of leprosy using multivariable negative binomial regression models for panel data with fixed-effects specification, crude and adjusted for relevant covariates.

As the outcome in this study was a rate (the new case detection rate of leprosy) negative binomial regression models were used as it is suitable for count data with overdispersion [46]. In these models, the rate is decomposed in a count using the logarithm of the population as an offset variable.

Longitudinal panel data models as used here include a disturbance (or error) term and a term for unmeasured time-invariant characteristics of each unit of analysis, such as quality of the municipality management and other sociocultural or historical characteristics of the municipalities.

Fixed-effect (FE) was used to control for the correlation between the time-invariant term with the coverage of the intervention under study, providing unbiased estimates of impact [47]. The Hausman specification test was used in order to confirm the appropriateness of the FE specification [48].

A total of 1,358 municipalities were selected to be included in the study. Seven municipalities without cases during the eight years of the study were not included in the model fitting because they had no cases and the fixed-effects model algorithms could not handle this [46], [48].

The analyses were performed using Stata version 10 [49]. The Ethics Committee in Research of Institute of Collective Health - Federal University of Bahia (protocol n ° 181.078), approved this study.

## Results

The selected 1,358 municipalities originate over 50% of the new leprosy cases detected each year in Brazil and the annual NCDR of leprosy decreased from 74.8 to 45.6 per 100,000 people over the study period from 2004 to 2011. This is a considerably higher reduction than in the total of the Brazilian municipalities (Table 1).

**Table 1:** Number of new cases and new case detection rate of leprosy in the Brazil and selected municipalities (n = 1,358), Brazil 2004–2011.

Year	Number of new cases - Selected municipalities (a)	Total number of new cases -Brazil (b)	% of cases the total of Brazil (a/b)	Leprosy new case annual detection rate* - Selected municipalities	Leprosy new case annual detection rate* - Brazil
**2004**	**30,024**	**50,565**	**59.3**	**74.8**	**28.2**
**2005**	**29,740**	**49,448**	**60.1**	**73.0**	**26.8**
**2006**	**26,908**	**43,642**	**61.6**	**65.1**	**23.3**
**2007**	**25,165**	**40,126**	**61.7**	**61.5**	**21.1**
**2008**	**24,816**	**39,047**	**63.5**	**58.8**	**20.5**
**2009**	**22,943**	**37,610**	**61.0**	**53.7**	**19.6**
**2010**	**21,469**	**34,894**	**61.5**	49.8	**18.2**
**2011**	**19,901**	**33,955**	**58.6**	**45.6**	**17.6**

*Per 100,000 inhabitants.

Table 2 shows that in the selected municipalities, during the study period, there was a marked expansion of the average municipal BFP coverage both in all population (from 24.6 to 44.7%) and in the target population (from 57.1 to 96.4%). There was also an increase in the mean municipality FHP coverage reaching 79.7% in 2011.

**Table 2:** Bolsa Família and Family Health Programs mean (standard deviation) coverage and the variables for selected municipalities, Brazil 2004–2011.

Variables	2004	2005	2006	2007	2008	2009	2010	2011	Percentage change 2004–11
**BFP coverage of the municipality population (%)**	**24.6 (14.2)**	**31.7 (16.2)**	**40.8 (18.0)**	**40.9 (17.2)**	**38.2 (16.7)**	**42.2 (16.8)**	**42.8 (17.3)**	**44.7 (19.1)**	**+81.7**
**BFP coverage of the target population (%)**	**57.1 (22.8)**	**75.1 (23.3)**	**92.7 (12.6)**	**95.6 (9.9)**	**94.4 (10.5)**	**96.7 (8.5)**	**96.7 (8.7)**	**96.4 (9.1)**	**+68.8**
**FHP coverage of the municipality population (%)**	**61.4 (34.6)**	**68.9 (31.1)**	**73.4 (28.9)**	**78.7 (26.3)**	**78.6 (25.4)**	**79.7 (24.5)**	**80.8 (23.9)**	**79.7 (24.4)**	**+29.8**
**Urbanization rate (%)**	**58.0 (21.2)**	**58.5 (21.1)**	**58.9 (21.0)**	**59.4 (20.8)**	**59.9 (20.8)**	**60.4 (20.7)**	**60.9 (20.6)**	**61.3 (20.6)**	**+5.6**
**Percentage of poor people in the municipality (%)**	**43.8 (18.7)**	**41.8 (18.4)**	**39.8 (18.2)**	**37.8 (18.0)**	**35.8 (17.8)**	**33.8 (17.7)**	**31.8 (17.6)**	**29.8 (17.6)**	**−31.9**
**Gini Index (0–1)**	**0.56 (0.05)**	**0.55 (0.05)**	**0.55 (0.05)**	**0.54 (0.05)**	**0.54 (0.05)**	**0.53 (0.05)**	**0.53 (0.06)**	**0.53 (0.06)**	**−5.3**
**Illiteracy rate (%)**	**23.1 (9.6)**	**22.6 (9.4)**	**22.0 (9.2)**	**21.4 (9.0)**	**20.8 (8.8)**	**20.2 (8.6)**	**19.6 (8.5)**	**19.6 (8.4)**	**−15.1**
**Unemployment rate (%)**	**9.0 (4.0)**	**8.7 (3.7)**	**8.4 (3.5)**	**8.1 (3.3)**	**7.8 (3.2)**	**7.5 (3.3)**	**7.2 (3.4)**	**6.9 (3.6)**	**−23.3**
**Average number of residents per household**	**3.9 (0.5)**	**3.9 (0.5)**	**3.8 (0.5)**	**3.7 (0.5)**	**3.7 (0.5)**	**3.6 (0.5)**	**3.6 (0.5)**	**3.5 (0.5)**	**−10.2**
**Percent of the population younger than 15 years (%)**	**34.7 (5.1)**	**34.7 (5.1)**	**34.7 (5.1)**	**30.5 (5.2)**	**30.0 (5.2)**	**29.5 (5.2)**	**28.3 (5.0)**	**28.3 (5.0)**	**−18.4**

BFP = *Bolsa Familia* Programme. FHP = Family Health Programme.

Marked improvements in the socioeconomic conditions was observed in the selected municipalities during the study period. The mean urbanization rate reached 61.3% in 2011. There were reductions in percentage of poor people in the municipality (from 43.8 to 29.8%), Gini Index (from 0.56 to 0.53), illiteracy rate (from 23.1 to 19.6%), unemployment rate (from 9.0 to 6.9%), average number of residents per household (from 3.9 to 3.5) and mean percentage population aged less than 15 years (from 34.7 to 28.3%).

Table 3 shows the crude and adjusted association between new case detection rate of leprosy with BFP and FHP coverage levels. Increase in BFP coverage exhibited a significant dose–response reduction in new case detection rate of leprosy, and the effect is maintained after the controlling for demographic and socio-economic variables. When compared with municipalities with low coverage, municipalities with intermediate, high and consolidated BFP coverage have significant reductions in the new case detection rate of leprosy in crude and adjusted models. For instance, reduction in municipalities with BFP consolidated coverage was 27% over the period (RR = 0.73; 95% CI  = 0.69–0.77) on the crude model and 21% in the model adjusted for selected covariates (RR = 0.79 95% CI  = 0.74–0.83).

**Table 3:** Fixed-effect negative binomial models for association between new case detection rate of leprosy and Bolsa Família Program and Family Health Program coverage, Brazil 2004–2011.

	New case detection rate of leprosy Risk Ratio (95 CI %)				
	BFP Models		FHP Models		BFP and FHP Model
	Crude	Adjusted	Crude	Adjusted	Adjusted
**BFP population coverage**					
**Low (0.0–27.75%)**	**1.00**	**1.00**	**-**	**-**	**1.00**
**Intermediate (27.76–48.10%)**	**0.86 (0.84–0.88)**	**0.90 (0.87–0.92)**	**-**	**-**	**0.89 (0.86–0.91)**
**High> = (48.11%)**	**0.83 (0.80–0.87)**	**0.87 (0.83–0.90)**	**-**	**-**	**0.85 (0.81–0.88)**
**Consolidated (>48.11% and TPC ≥100% for at least 4 years)**	**0.73 (0.69–0.77)**	**0.81 (0.77–0.85)**	**-**	**-**	**0.79 (0.74–0.83)**
**Family Health Programme Coverage^‡^**					
**1 st tertile (0–72.02%)**	**-**	**-**	**1.00**	**1.00**	**1.00**
**2 st tertile (72.03–95.06%)**	**-**	**-**	**0.99 (0.96–1.02)**	**1.02 (0.99–1.05)**	**1.05 (1.02–1.09)**
**3 st tertile (Over 95.06%)**	**-**	**-**	**1.04 (0.99–1.08)**	**1.09 (1.05–1.13)**	**1.12 (1.08–1.17)**
Illiteracy rate> = 20.42% *	**-**	**1.12 (1.07–1.18)**	**-**	**1.14 (1.08–1.20)**	**1.12 (1.07–1.18)**
Gini Index> = 0.54 *	**-**	**1.07 (1.03–1.11)**	**-**	**1.07 (1.03–1.11)**	**1.07 (1.04–1.11)**
Unemployment rate> = 7.47% *	**-**	**1.19 (1.16–1.23)**	**-**	**1.20 (1.16–1.23)**	**1.20 (1.16–1.23)**
Urbanization rate> = 59.8% *	**-**	**0.99 (0.93–1.06)**	**-**	**1.01 (0.94–1.07)**	**0.99 (0.93–1.06)**
Average number of residents per household> = 3.6 *	**-**	**1.04 (1.01–1.07)**	**-**	**1.05 (1.02–1.09)**	**1.04 (1.01–1.08)**
Percent of the population younger than 15 years> = 31.1% *	**-**	**1.11 (1.07–1.14)**	**-**	**1.13 (1.10–1.17)**	**1.12 (1.08–1.15)**
**Percentage of poor people in the municipality> = 27,42%**	**-**	**1.13 (1.09–1.18)**	**-**	**1.14 (1.09–1.19)**	**1.13 (1.08–1.18)**
**Number of observations**	**10,808**	**10,808**	**10,808**	**10,808**	**10,808**
**Number of municipalities**	**1,351**	**1,351**	**1,351**	**1,351**	**1,351**

Data are risk ratio (95% CI) unless otherwise specified. TPC = target population coverage. ^‡^Cutoff taken from tertiles of the distribution of FHP coverage of the total population. *Cutoff is median value.

**The regression model to be estimated was as follows: Yit =  αi + β1BFPit + β2FHPit + βnXnit + uit**

Where *Yit* was the leprosy detection rate for the municipality *i* in year *t, αi* is the fixed effect for the municipality *i* that captures all unobserved time-invariant factors, *BFPit* is the Bolsa Familia Program coverage for the municipality *i* in the year *t, FHPit* the Family Health Program coverage for the municipality *i* in the year *t, Xnit* was the value of each *n* covariate of the model with in the municipality *i* in the year *t*, and *uit* was the error.

The analysis shows a significant increase in NCDR of leprosy as FHP coverage increases. In the adjusted model, compared with the low tertile of FHP coverage, in the medium tertile of FHP coverage (72.03–95.08%) there was an increase of 5% over the period (RR  = 1.05 95% CI  = 1.02–1.09) and for the higher tertile and increase of 12% over the period (RR  = 1.12 95% CI  = 1.08–1.17).

All selected covariates except urbanization rate were significantly associated with the new case detection rate of leprosy.

## Discussion

This is the first evidence of the join impact of a conditional cash transfer and of a primary health care programmes on the incidence/detection of leprosy. BFP was associated with significant reduction in the NCDR of leprosy, and FHP was associated with significant increase in the NCDR of leprosy. Both effects were statistically significant and showed a dose-response effect.

We postulate that the first effect - reduction in new case detection rate with the BFP - reflects a reduction in incidence of leprosy, consistent with the cash transfer component of BFP leading to improving living conditions. Poverty itself is a determinant of leprosy [9], [10], [11]; cash transfer reduces not only poverty but also specific aspects of poverty associated with leprosy, like inequality [9], undernutrition and food shortage [9], [10], [11]. There is consistent evidence that conditional cash transfer programs increase food expenditure [50], [51], [52], [53]. In Brazil, BFP increased access to food and improved food quality and diversity [53], [54].

The second finding was an increase in new case detection rate of leprosy associated with the FHP coverage. We postulate that this reflects not a genuine increase in incidence, but an increased detection of cases that would otherwise remain undiagnosed - the hidden prevalence. FHP increases contact of individuals to health services and therefore is likely to facilitate self-reporting and diagnosis of leprosy cases in primary health care units. Other studies in Brazil showed increased coverage of primary health care contributing to an increase in new case detection rate of leprosy [35], [55], [56].

In Brazil leprosy has been a nationally notifiable disease for many decades. Brazil has a single surveillance information system. Each reported case is included in the database of the secretary of health of the municipalities and transmitted to the Ministry of Health. The NCDR depends of the capacity of health facilities identify the signs and symptoms of leprosy for diagnosis. Treatment was decentralized offering health care in a larger number of municipalities [3], [35]. The National Leprosy Control Programme recommends treatment with multidrug therapy (MDT) according to World Health Organization recommendation and distributes it free of charge. The amount of MDT blister packs needed is estimated based on reported data, which guarantees an approximate relation between cases reported and cases treated [57].

Although better detection leads to a short-term increase in the NCDR, we fully expect that better detection will eventually lead to a long term reduction in incidence, as a result of lower number of infectious cases due to reduced hidden prevalence and earlier diagnosis and treatment of clinical cases, identification of contacts and better outcome of treatment [55], [56].

Social interventions can have an impact on the leprosy transmission or clinical disease progression. The mean incubation period of leprosy is 2–5 years, but can be as long as 20 years [11]. Therefore would be necessary to analyze a longer period to infer whether the BFP and FHP had an impact on the transmission of leprosy.

As our inference level is ecologic - we want to determine the effectiveness of social and health policy at an aggregate level – we do not commit ecological fallacy. The ecologic design also allows measurement of the effect of externalities of the BFP, which can represent an important part of its global effect [40]: the relief from poverty of a relevant proportion of the population in a small municipality can make the local economy grow, and families that are not recipients of the program are going to benefit from this spill-over effect. Furthermore, leprosy affects mainly the poor and extremely poor individuals and many of them are eligible for BFP and live in deprived areas where FHP is priority implemented.

We used municipality as the unit of analysis because the National Social Assistance System (SUAS) and National Health System (SUS) are decentralized in Brazil and BFP and FPH were implemented at the municipality level [19], [29], [32].

Moreover, the application of a sophisticated statistical methodology allowed us to analyze a time series for each municipality in the data set. Negative binomial regression of panel data, widely used in econometric literature, has recently been introduced in health studies [36], [37], [39], [40]. Panel data essentially defined a time series analysis for each municipality and contrasted the trends between them, making this a more rigorous approach than a simple purely cross-sectional data [48].

We used a coverage indicator combining BFP coverage of the total population of the municipality and BFP coverage of the target population (poor and extremely poor). We did this to estimate the “spill over” effect of the BFP on inhabitants of the municipality that were not enrolled in the programme [40].

Additionally, because leprosy is a highly focused disease in some regions of Brazil, only municipalities located in areas with high disease burden were included in the analysis. Therefore, the results can not be generalized to municipalities in areas of low prevalence of leprosy.

Leprosy clusters were formed by different groups of neighboring municipalities. Some municipalities in these clusters had lower case detection rates than the average case detection rate in Brazil. It is possible that fewer cases were detected because of limitations of the healthcare system, such as low population coverage and the inability of healthcare professionals to diagnosis leprosy. Municipalities with a low detection rate that are located in high-risk areas have to intensify case finding and treatment [3].

Another possible limitation was that the annual values of sociodemographic variables were obtained from linear interpolation and extrapolation from decennial census data. Since we did not expect substantive changes in these trends is unlikely that these estimates introduced any significant source of error. However, the categorization of variables can limit the possible bias introduced by the techniques of crude interpolation by smoothing sharp fluctuations artificially introduced by the method.

Making socioeconomic covariate data at the municipal level available for inclusion in multivariate analyses strengthens the case for the effectiveness of health programs. This is particularly important for the case of Brazil and several other countries in Latin America, where the expansion of health services in the last decade has occurred simultaneously with other forms of social progress, such as improvements in sanitation infrastructure, educational attainment, and economic development [58].

We did not think it necessary to include in the model a variable representing time as in our view any secular trend was controlled by the use of rate ratios, contrasting different groups of coverage changes according to the same time trends. Moreover relevant confounding factors, which could have been represented by an artificial time variable, have been included in the models, and the individual-specific term of the fixed effects model control for time-invariant unobserved confounding variables [48]. Sensitivity analysis showed that the introduction of a time variable created an over specification problem in the models.

One of the many strengths the study is that expansion of BFP and the FHP at different rates in the Brazilian municipalities in recent decades created the opportunity to investigate their effects on new case detection rate of leprosy. Despite the limitations, the results of this study are consistent and illustrate the contribution FHP in improving diagnosis and therefore of the control of leprosy. It also point for a positive effect of the BFP cash transfer in reducing leprosy, confirming the contribution of the social determinants to leprosy control.

The conditional cash transfer programs has steadily increased around the world, including in leprosy endemic countries located in Africa and Asia, such as Nigeria and Indian [2], [21], [22]. Conditional cash transfer programs are one way to boost demand and reduce barriers to access for health services particularly in primary health care units to poor and extremely poor individuals. Thus, it is necessary an effective primary health care in these populations able to comply with basic health needs and have attending conditions required by the conditional cash transfer programs in these countries.

Given the expansion of cash transfer programs and their relevance to public health it is necessary to accumulate evidence of mechanisms and pathways through which cash transfers affect epidemiologically related factors leprosy and other poverty related disease.

Social interventions, such as conditional cash transfer programs for the poorest groups, improvements in health care, and progress in social and environmental determinants are essential for the control of poverty related infectious diseases and in particular leprosy [59]. It is expected that these results contribute with arguments to the discussion on the relationship between distributive social policies, primary health care and health conditions of the population in developing countries worldwide.
